# Critical slowing in a Hodgkin-Huxley neuron near spiking threshold

**DOI:** 10.1186/1471-2202-13-S1-P34

**Published:** 2012-07-16

**Authors:** Alex Bukoski, D Alistair Steyn-Ross, Moira L Steyn-Ross

**Affiliations:** 1 College of Veterinary Medicine, University of Missouri, Columbia, MO 65203, USA; 2 School of Engineering, University of Waikato, Hamilton 3240, New Zealand

## 

**Figure 1:**
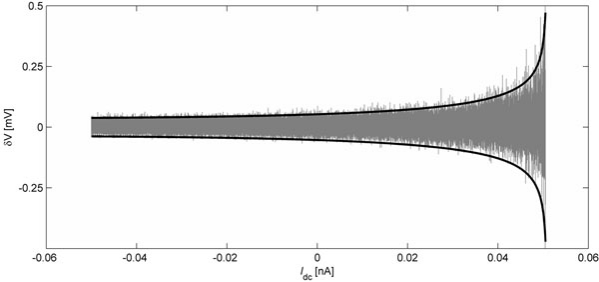
Subthreshold response to white-noise perturbation as a function of *I*_dc_ stimulus current. Solid black curves show theoretical ±3 standard deviation limits for voltage excursions δV away from equilibrium and each vertical gray trace shows actual maximal excursions recorded in 200 ms stochastic simulation runs of the four-variable Hodgkin-Huxley equations at each of 2000 settings for stimulus current ranging from -0.05 to +0.05 nA.

As noted by Freeman [1], a quiescent neuron approaching spiking threshold exhibits a nonlinearly increasing sensitivity to stimulus. This growth of subthreshold susceptibility can be quantified by applying a linear multivariate Ornstein-Uhlenbeck analysis to the neuron equations, and this has been verified recently [2] for a reduced two-variable spiking model due to Wilson [3]. Here we generalize this stochastic analysis to the classical four-variable conductance-based Hodgkin-Huxley neuron with type-I excitability [4], perturbed by independent white noises entering the drive current and gating variables. We demonstrate critical slowing down—growth in amplitude simultaneous with decay in frequency of soma voltage fluctuations—as the neuron approaches firing threshold. We show that this behavior is a direct result of the interaction between the model’s eigenvalue structure and the noisy environment in which a biological neuron is presumed to function. Stochastic calculus results applied to this four-variable system predict fractional power-law scaling in the divergences for both voltage fluctuations (see Fig. 1) and correlation time as the critical point of saddle-node annihilation is closely approached. Such divergences are expected to be universal characteristics for all type-I neuron models. If these critical fluctuations are communicated to neighboring neurons via ubiquitous electrical gap junctions, then subthreshold neuronal dynamics may play an important role in overall cortical dynamics.
